# Serum Levels of TNF-*α*, IL-12/23p40, and IL-17 in Plaque Psoriasis and Their Correlation with Disease Severity

**DOI:** 10.1155/2014/467541

**Published:** 2014-12-21

**Authors:** Aikaterini Kyriakou, Aikaterini Patsatsi, Timoleon-Achilleas Vyzantiadis, Dimitrios Sotiriadis

**Affiliations:** ^1^2nd Dermatology Department, Aristotle University School of Medicine, General Hospital “Papageorgiou”, Nea Efkarpia Ring Road, 564 03 Thessaloniki, Greece; ^2^Department of Microbiology, Aristotle University School of Medicine, 541 24 Thessaloniki, Greece

## Abstract

A case-control study was performed to assess the serum levels of TNF-*α*, IL-12/23p40, and IL-17 in patients with plaque psoriasis, compare them with healthy controls, and correlate them with disease severity, as represented by Psoriasis Area Severity Index (PASI). 32 consecutively selected, untreated patients with active, chronic plaque psoriasis were recruited and compared to 32 age- and sex-matched healthy controls. Serum cytokine levels were determined by solid phase sandwich enzyme linked immunosorbent assay (R&D Systems Europe, Ltd.). The mean serum levels of TNF-*α* were significantly higher in psoriatic patients compared to those of controls (Mann-Whitney *U* test; *P* = 0.000). However, the median serum levels of neither IL-12/23p40 nor IL-17 differ significantly between the 2 groups (Mann-Whitney *U* test; *P* = 0.968 and *P* = 0.311, resp.). No significant correlations were found between PASI and any of the cytokine serum levels (Spearman's rank test; *P* > 0.05). Despite the well-evidenced therapeutic efficacy of biologic agents targeting TNF-*α*, IL-12/23p40, and IL-17, serum levels of TNF-*α*, IL-12/23p40, and IL-17 do not seem to correlate with the severity of psoriatic skin disease in untreated patients, as represented by PASI. Further investigation may add more data on the pathogenetic cascade of psoriasis.

## 1. Introduction

Psoriasis is a common immune-mediated inflammatory disease that affects the skin, joints, and nails. Its pathogenesis is a complex interaction among genetic, immunological, and environmental components [1]. While pathogenesis of psoriasis has become better understood, tumor necrosis factor-*α* (TNF-*α*), IL-12, IL-23, and IL-17 seem to be critical checkpoints of psoriatic inflammation [1, 2]. Therefore, treatment of psoriasis has been revolutionized by targeting these inflammatory cytokines as key drivers of disease pathogenesis. Anti-TNF-*α* agents (adalimumab, etanercept, infliximab), anti-IL12/23p40 agent (ustekinumab), and upcoming agents targeting IL-17 and its receptor (secukinumab, ixekizumab, brodalumab) seem to be effective in patients with moderate to severe chronic plaque psoriasis [3–18]. However, the inconsistent response to therapy for some patients remains a hindrance. Therefore, there is still a need to expand knowledge of psoriasis etiopathogenesis.

The main aim of this study was to assess the serum levels of TNF-*α*, IL-12/23p40, and IL-17 in patients with plaque psoriasis, compare them with healthy controls, and correlate them with the disease severity.

## 2. Materials and Methods

A case-control, hospital-based study was performed in which 32 consecutively selected psoriatic patients were recruited and compared to 32 age- and sex-matched healthy controls. Subjects above the age of 18 years with clinically diagnosed active, chronic plaque psoriasis, who had not received any systematic antipsoriatic treatment or any topical treatment against cutaneous or nail psoriasis for at least a year, were eligible to participate in this study. Exclusion criteria were being erythrodermic, pustular, or palmoplantar or having other forms of psoriasis, nail psoriasis, psoriatic arthritis, skin conditions and treatments at baseline, that would interfere with psoriasis evaluation, immunosuppression, malignancies, autoimmune/genetic/metabolic/rheumatic diseases, and bacterial, viral, or fungal infection up to 4 weeks prior to inclusion in the study. The control group was composed of healthy volunteers with no family history of psoriasis and no history of having received medication of any kind during the 3 weeks prior to the blood sample. All controls were recruited from visitors and employees of our hospital.

The diagnosis of plaque psoriasis was confirmed in all cases by 2 dermatologists based on established clinical criteria [19]. Patients' age, gender, age at onset of psoriasis, skin psoriasis duration, family history of psoriasis, Psoriasis Area Severity Index (PASI) (0–72) [20], and serum cytokine levels of TNF-*α*, IL-12/23p40, and IL-17 were recorded. PASI was assessed by one, specific dermatologist of the research group. Ethics board approval and written informed consent from all patients were obtained.

Venous blood samples (5–10 mL) of all patients were collected between 09:00 and 11:30 a.m. in vacutainer tubes, without anticoagulant, under sterile conditions. After samples were rapidly centrifuged, serum was obtained and immediately stored at −70°C until batch processed. All samples were tested once per patient. Serum cytokine levels of TNF-*α*, IL-12/23p40, and IL-17 were determined by solid phase sandwich enzyme linked immunosorbent assay (R&D Systems Europe, Ltd.). All assays were conducted according to manufacturers' protocols. These assays only detected human cytokines and as per manufacturers' protocols, the minimum detectable dose was 1.6 pg/mL for TNF-*α* and typically less than 15 pg/mL for IL-12p40 and IL-17.

The objectives of this study were (1) to assess the serum levels of TNF-*α*, IL-12/23p40, and IL-17 in patients with plaque psoriasis, (2) to detect possible statistically significant differences in the serum levels of TNF-*α*, IL-12/23p40, and IL-17 between the psoriatic patients and healthy controls, and (3) to evaluate the correlations of the serum levels of TNF-*α*, IL-12/23p40, and IL-17 with the disease severity as indicated by PASI.

It is of note that serum levels of TNF-*α*, IL-12/23p40, and IL-17 may potentially be elevated due to psoriatic skin, joint, or nail disease, while PASI is an indicator of the severity of skin disease.* Therefore, this study was designed to include only patients having exclusively skin lesions.* Moreover, psoriatic patients with nail involvement and/or psoriatic arthritis are reported to have more severe psoriasis, as assessed by PASI, compared to those without [21–24]. Consequently, a relatively mild median PASI is likely in our sample.

### 2.1. Statistical Analysis

Statistical analysis of the data was performed using the Statistical Package for Social Sciences (SPSS), version 22.0 (SPSS, Inc., Chicago, IL). Descriptive statistics were used to describe the study population's characteristics. Shapiro-Wilk test was used to test the normality of continuous variables. All continuous variables were expressed as the mean ± standard deviation or median (range) for normally or nonparametric distributed numeric values, respectively. Frequency distributions and percentages were used for categorical data. Fisher's exact test was used to compare dichotomous variables between psoriatic patients and controls, while Mann-Whitney *U* test was used to compare continuous variables. Spearman's rank test was also used to explore relationships between continuous variables. Multiple linear regression was fitted using PASI as the outcome variable and the different demographic and clinical characteristics measured as the covariate variables. All tests were two sided, and the significance level was chosen to be *α* = 0.05.

## 3. Results and Discussion

A total of 32 psoriatic patients, 9 males (28.12%) and 23 females (71.87%), and 32 healthy control subjects were included in the study (Table 1). No statistical difference was observed between psoriatic patients and control subjects for gender and age (*P* > 0.05). The mean age of the included psoriatic patients was 44.53 ± 15.60 years, the mean age at onset of psoriatic skin manifestations was 34.38 ± 17.62 years, and the median duration of skin disease was 11.00 (range: 1.00–25.00) years. Of the 32 included patients, 6 (18.75%) had a family history of psoriasis. The median PASI was 4.10 (range: 2.20–15.00). Psoriatic patients' and controls' serum levels of TNF-*α*, IL-12/23p40, and IL-17 are presented in Table 2.

Equal incidence of psoriasis in males and females has been reported worldwide. However, the limited available data regarding Greek population shows a female predominance [25]. This is confirmed by the present study, but may be explained by a selection bias. Gender seems to influence quality of life (QoL) in psoriatic patients, as women score higher on QoL scores than men [26]. It may be speculated that women are more worried than men about their disease and seek treatment directly; thus, female predominance has been reported in this study.

The median serum levels of TNF-*α* were significantly higher in psoriatic patients than in the controls (Mann-Whitney *U* test; *P* = 0.000). However, neither the median serum levels of IL-12/23p40 nor IL-17 differ significantly between the 2 groups (Mann-Whitney *U* test; *P* = 0.968 and *P* = 0.311, resp.). No significant correlations were found between PASI and any of the cytokine serum levels (Table 3). We also tried to fit a multivariable linear regression model using PASI as the dependent variable and the other demographic and clinical characteristics as the independent ones. The multiple linear regression model showed that none of the covariates were statistically significant in predicting PASI (Table 4).

Psoriasis is an immune-mediated inflammatory disease that affects the skin, joints, and nails. In genetically predisposed individuals, all the elements of the epidermis and the dermis that are involved in the maintenance of the barrier integrity are deregulated in response to either an environmental or self-antigenic insult [1]. Apart from the cellular components involved in psoriasis, mainly keratinocytes and T lymphocytes, the cytokines produced by the main Th subsets play a determining role in its pathogenesis [1]. Despite the fact that some progress has been made to better understand their precise mechanism of action in psoriasis, there is still much to be investigated.

In the literature, several studies have been conducted to evaluate the levels of various circulating cytokines in the serum of psoriatic patients and compared the results with those in healthy controls [27–36]. In line with our results, most studies have reported that the serum levels of TNF-*α* are significantly increased in patients with psoriasis compared with those of healthy controls [27–29, 32–34, 36–38]. However, Tigalonova et al. [35] and Jacob et al. [31] have found that the serum levels of TNF-*α* do not significantly differ between psoriatic patients and controls.

There has been no consistent conclusion in the literature on the correlation between the serum levels of TNF-*α* and disease severity, as assessed by PASI. Our study detected no significant correlations between the PASI and the TNF-*α* serum levels. There are papers confirming no correlation between TNF-*α* and PASI [28, 29, 37], and others stating the opposite [32, 34]. This controversy may be due to the heterogeneity of the inclusion criteria and study populations (Table 5).

Our study also showed that the median serum levels of IL-12/23p40 did not significantly differ between psoriatic patients and controls and did not correlate with PASI. The serum levels of IL-12/23p40 in patients with psoriasis are poorly investigated. To our knowledge, the only study in which the serum levels of IL-12/23p40 in psoriatic patients and controls were assessed has been conducted by Arican et al. [29]. Contrary to our results, Arican et al. reported that the levels of IL-12/23p40 were significantly elevated in the serum of psoriatic patients compared to controls and correlated significantly with PASI [29].

On the other hand, the levels of IL-12p70 have been more extensively investigated and found to be significantly elevated in the serum of psoriatic patients compared to those of controls [30, 33, 34], with the exception of Jacob et al. who reported decreased levels of IL-12p70 in the sera of psoriatic patients [31]. Moreover, it has been reported that serum levels of IL-12p70 correlate with PASI [34].

Regarding the serum levels of IL-17 in psoriasis, results reported in the literature are controversial. In accordance with our results, most studies presented no significant difference in the serum levels of IL-17 between psoriasis and control groups [29, 39, 40]. On the other hand, Takahashi et al. [34] and El-Moaty Zaher et al. [41] have recently reported significantly increased levels of IL-17 in the sera of psoriatic patients compared to controls. In line with El-Moaty Zaher et al. [41] but contrary to other reported literature [29, 34, 40], we suggested that high levels of IL-17 do not correlate with PASI. However, it is noteworthy that all papers, with the exception of Takahashi et al. [34], reported significant correlation between IL-17 and PASI, although no difference was detected in the serum levels of IL-17 between psoriatic patients and controls.

Clearly, there is some disagreement concerning the results on the correlation between the serum levels of TNF-*α*, IL-12/23p40, and IL-17, and the disease severity, as assessed by PASI. This controversy is probably due to the heterogeneity of the inclusion criteria and study populations (Table 5). The serum levels of TNF-*α*, IL-12/23p40, and IL-17 may potentially be elevated due to psoriatic skin, joint, or nail disease, while PASI is an indicator of the severity of skin disease. When psoriatic patients with concomitant joint and/or nail disease are included, it remains uncertain if the differences found on the serum levels of TNF-*α*, IL-12/23p40, and IL-17 can be explained solely by the presence of cutaneous psoriasis, psoriatic arthritis, nail psoriasis, or a combination of factors. Moreover, under this spectrum, the correlation found between the indicator of the severity of skin disease (PASI) and any of the cytokines' serum levels might also be deceptive.* Therefore, we strongly believe that the most valid results arise when only patients having exclusively skin lesions are included in the respective analyses*.

Finally, we would like to discuss some limitations of PASI, which may be considered to have influenced our results. PASI has been criticized for being resource intensive, being complex, lacking sensitivity, being low in accuracy, and having a nonlinear scale [42, 43]. Due to the fact that PASI is not a linear scale, improvements in PASI score do not linearly reflect improvement in psoriasis [44]. Moreover, it lacks sensitivity at the lower end of its range and the upper half of its range is redundant [42]. However, PASI score is the most commonly used clinical measure in research, the most extensively studied psoriasis clinical severity score, and the most thoroughly validated according to methodological validation criteria [42, 43]. Therefore, despite its limitations, PASI score has been recommended for the scientific evaluation of the clinical severity of psoriasis [42].

## 4. Conclusions

Despite the well-evidenced therapeutic efficacy of biologic agents targeting TNF-*α*, IL-12/23p40, and IL-17, our results showed that the serum levels of TNF-*α*, IL-12/23p40, and IL-17 do not seem to correlate with the severity of psoriatic skin disease in untreated patients, as represented by PASI. Moreover, it is of interest that the serum levels of TNF-*α* were significantly higher in psoriatic patients compared to those of controls, contrary to those of IL-12/23p40 and IL-17 that did not significantly differ between the 2 groups. Based on our findings (especially for TNF-*α*), we can assume that what happens in the skin is not always reflected in the blood and vice versa. Serum cytokine levels do not necessarily reflect local regional disease activity [34]. The serum cytokine concentrations may be altered by several processes like the production, tissue deposition, degradation, and elimination of these molecules [29]. The origin of circulating cytokines in blood serum in psoriatic patients is still not completely clear. Huge amounts of free cytokines are required, to achieve the cytokine concentration that can induce biological responses at distant skin lesions [29]. It appears likely that changes in the cytokine serum levels of psoriatic patients may not be the cause, but the consequence of the disease. Maybe, there are sources other than the skin that contribute to the production of these cytokines; this theory may provide a potential mechanism linking psoriasis with its extracutaneous comorbidities [37]. Further investigation may add more data on the pathogenetic cascade of psoriasis. Additionally, well-designed studies will contribute to clearly state what the real relationship is between the serum levels of various inflammatory molecules and PASI and whether the serum levels of these cytokines may be used as an objective parameter for psoriasis activity and clinical severity.

## Figures and Tables

**Table 1:** Patients' demographic and clinical characteristics, as well as cytokine serum levels.

Psoriatic patients (*n* = 32)										Controls (*n* = 32)		
Patient number	Age (years)	Gender	Age at onset (years)	Disease duration (years)	Family history	PASI (0–72)	TNF-*α* (pg/mL)	IL-12/23p40 (pg/mL)	IL-17 (pg/mL)	TNF-*α* (pg/mL)	IL-12/23p40 (pg/mL)	IL-17 (pg/mL)
1	72.00	Female	71.00	1.00	No	15.00	7.69	198.34	0.00	5.41	72.08	0.00
2	45.00	Female	22.00	13.00	No	6.40	7.69	50.02	2.56	1.80	103.33	1.42
3	43.00	Female	21.00	22.00	No	2.20	7.71	185.86	6.28	5.57	35.75	1.42
4	57.00	Male	52.00	5.00	No	6.20	7.37	59.26	8.59	4.56	103.33	11.35
5	62.00	Female	47.00	15.00	No	5.40	7.40	142.79	4.75	3.53	44.86	0.00
6	42.00	Female	41.00	1.00	No	2.80	7.06	62.68	8.59	2.68	112.40	0.00
7	52.00	Female	50.00	2.00	No	2.20	7.69	116.88	0.00	1.96	51.69	0.00
8	40.00	Male	25.00	15.00	No	2.20	10.80	51.74	0.00	5.96	207.51	1.42
9	23.00	Male	13.00	10.00	Yes	2.30	5.66	45.18	0.00	7.13	93.25	0.00
10	23.00	Female	10.00	13.00	No	4.10	2.34	48.36	0.45	1.98	150.20	0.00
11	30.00	Female	10.00	20.00	Yes	6.30	4.77	59.71	0.45	3.84	65.02	0.00
12	71.00	Female	69.00	2.00	No	14.90	6.63	196.34	0.00	4.41	34.86	0.00
13	23.00	Male	22.00	1.00	No	9.40	7.37	60.33	1.96	2.80	110.38	0.00
14	37.00	Male	24.00	13.00	No	3.70	7.03	152.85	8.45	4.57	98.29	0.00
15	53.00	Female	49.00	4.00	Yes	5.20	10.60	56.27	8.58	4.66	166.01	0.00
16	62.00	Female	60.00	2.00	No	7.40	10.40	114.79	3.18	5.83	94.25	5.67
17	29.00	Female	16.00	13.00	No	5.30	7.74	38.68	7.80	3.28	136.36	9.49
18	69.00	Male	44.00	25.00	No	3.20	7.37	177.88	1.00	2.19	97.28	1.80
19	34.00	Female	25.00	9.00	Yes	3.10	2.23	60.74	0.00	7.10	73.09	4.26
20	25.00	Male	21.00	4.00	No	5.10	7.71	56.18	0.00	7.13	39.70	2.60
21	67.00	Female	56.00	11.00	No	5.20	8.37	51.36	0.00	2.10	26.65	0.00
22	34.00	Male	32.00	2.00	Yes	2.20	7.17	43.71	0.00	6.99	36.00	0.00
23	56.00	Female	51.00	5.00	Yes	15.00	5.23	174.34	0.00	3.84	75.10	0.00
24	29.00	Female	18.00	11.00	No	5.30	7.40	44.02	0.26	3.57	138.34	2.84
25	49.00	Female	36.00	13.00	No	2.20	10.01	181.70	8.59	3.05	146.74	2.13
26	33.00	Female	16.00	17.00	No	2.20	7.69	43.27	8.57	3.84	27.89	4.96
27	61.00	Female	58.00	3.00	No	3.80	10.80	180.79	3.19	6.98	127.30	0.00
28	47.00	Female	36.00	11.00	No	3.10	7.66	75.68	0.00	2.98	91.50	0.00
29	23.00	Male	19.00	4.00	No	4.10	7.41	139.88	2.00	2.67	87.40	0.00
30	54.00	Female	32.00	22.00	No	3.90	6.90	48.74	0.00	2.12	142.20	4.14
31	49.00	Female	36.00	13.00	No	4.20	7.40	36.18	0.00	3.23	113.40	2.30
32	31.00	Female	18.00	13.00	No	2.20	9.10	41.60	0.28	7.10	26.10	1.80

**Table 2:** Serum levels of TNF-*α*, IL-12/23p40, and IL-17 in psoriatic patients and controls.

Cytokines	Psoriatic patients (*n* = 32)	Controls (*n* = 32)	*P* values
TNF-*α* (pg/mL)			
Mean ± SD	7.45 ± 1.98	4.21 ± 1.80	0.000^*^
Median (min–max)	7.41 (2.23–10.80)	3.84 (1.80–7.13)	
IL-12/23p40 (pg/mL)			
Mean ± SD	93.63 ± 58.20	91.51 ± 45.89	0.968
Median (min–max)	60.02 (36.18–198.34)	93.75 (26.10–207.51)	
IL-17 (pg/mL)			
Mean ± SD	2.67 ± 3.46	1.80 ± 2.81	0.311
Median (min–max)	0.45 (0.00–8.59)	0.00 (0.00–11.35)	

Mann-Whitney *U* test; ^*^statistically significant.

**Table 3:** Correlation between PASI and cytokines levels evaluated by the Spearman rank correlation.

Correlations	Statistics	Serum levels of TNF-*α*	Serum levels of IL-12/23p40	Serum levels of IL-17
PASI score	Spearman's Rho	−0.174	0.167	−0.035
	*P* value	0.340	0.360	0.849

**Table 4:** Results of multiple linear regression analysis for PASI.

Variables	Unstandardized coefficients		Standardized coefficients	t	*P* value	95.0% confidence interval for *B*	
	B	Std. error	Beta			Lower bound	Upper bound
(Constant)	6.038	3.544		1.704	0.102	−1.294	13.370
Age	0.339	0.317	1.453	1.073	0.295	−0.315	0.994
Gender	0.523	1.303	0.066	0.402	0.692	−2.171	3.218
Psoriasis onset	−0.265	0.315	−1.281	−0.841	0.409	−0.917	0.387
Duration	−0.483	0.330	−0.917	−1.464	0.157	−1.165	0.199
Family history	−0.150	1.519	−0.016	−0.099	0.922	−3.291	2.992
TNF-α	−0.520	0.335	−0.283	−1.552	0.134	−1.212	0.173
IL-12/IL-23p40	0.016	0.012	0.259	1.377	0.182	−0.008	0.041
IL-17	−0.145	0.169	−0.138	−0.859	0.399	−0.495	0.205

	*R*	*R* ^2^	Adjusted *R* ^2^	Std. error of the estimate			*P* value

Model summary	0.700	0.489	0.312	3.02314			0.027^*^

Dependent variable: PASI; predictors: (constant), age, gender, age at psoriasis onset, duration, family history, TNF-α, IL-12/IL-23p40, and IL-17; ^*^statistically significant.

**Table 5:** Inclusion criteria and populations' characteristics of the published studies conducted to evaluate the levels of various circulating cytokines in the serum of psoriatic patients.

Ref. number	Study populations' characteristics/inclusion criteria						
	Age (years)	Sex F/M	Clinical forms of skin psoriasis included	Psoriatic arthritis (PsA)	PASI (0–72)	Nail psoriasis	Treatment
[27]	N/A	N/A	Psoriatic patients; not further clarified	N/A	N/A	N/A	Untreated topically and systematically ≥14 days before enrolment

[28]	Mean ± SD: 40.2 ± 17.4 Range: 6.0–72.0	31/29	Plaque psoriasis 83.3%, guttate 10.0%, flexural 3.3%, pustular 1.6%, palmoplantar 1.6%	Present in 23.3%	PASI ≤ 25: 76.6% PASI > 25: 23.3%	N/A	Untreated topically ≥2 weeks before enrolment Untreated systematically ≥6 weeks before enrolment

[29]	Mean ± SD: 35.0 ± 15.5 Range: 7.0–79.0	12/18	Plaque psoriasis	Excluded	Mean ± SD: 9.3 ± 8.15 Range: 1.5–33.3	N/A	Untreated topically and systematically ≥2 months before enrolment

[30]	Mean: 38.0 Range: 18.0–77.0	20/35	Psoriatic patients; not further clarified	N/A	Mean ± SD: 21.7 ± 8.3	N/A	N/A (treatment before enrolment)

[31]	N/A	N/A	Plaque psoriasis: 9/12 Guttate psoriasis: 2/12 Erythrodermic psoriasis: 1/12	N/A	N/A	N/A	10/12 untreated at the time of enrolment 2/12 on methotrexate

[32]^*^	Median: 52.5 Range: 18.0–81.0	25/12	Plaque psoriasis	N/A	Median: 11.4 Range 3.5–42.0	N/A	N/A

[33]	N/A	14/31	Plaque psoriasis	N/A	N/A	N/A	N/A

[34]	Mean ± SD: 47.5 ± 7.6 Range: 25.0–72.0	41/81	Psoriasis vulgaris: 102/122 Guttate psoriasis: 7/122 Erythrodermic psoriasis: 5/122	Present in 8/122	Mean ± SD: 7.3 ± 4.2 Range: 0.7–32.3	N/A	Treated, untreated, well-controlled, and poorly controlled cases. Treatments: topical steroid, topical vitamin D3, psoralen ultraviolet A, and systemic treatments (etretinate, ciclosporin)

[35]	Stable, plaque type psoriasis: Mean: 50.0 Range: 24.0–85.0 Highly active psoriasis: Mean: 47.7 Range: 27.0–76.0 Acute guttate psoriasis: Mean: 31.3 Range: 18.0–49.0	N/A	Stable, plaque type psoriasis: 16/52 Highly active psoriasis: 22/52 Acute guttate psoriasis: 14/52	N/A	N/A	N/A	None of the patients received local nor systemic antipsoriatic treatment prior to the study

[36]	Mean ± SD: 34.5 ± 13.3 Range: 15.0–65.0	13/17	Psoriasis vulgaris: 90%; 10% not clarified	4/30: joint complaints 1/4: showed radiological evidence of PsA	N/A	N/A	N/A

[37]	Mean: 47.0 Range: 21.0–64.0	4/10	Psoriasis vulgaris	Excluded	Mean: 8.5 Range: 2.0–25.3	N/A	Untreated, topically and systematically ≥4 weeks before enrolment

[38]	Median: 52.5 Range: 15.0–82.0	19/1	Plaque psoriasis: 18/20 Suberythrodermic psoriasis: 1/20 Pustular psoriasis: 1/20	N/A	Median: 11.4 Range: 3.0–40.5	N/A	Untreated topically and systematically ≥10 days before enrolment

[39]	Mean ± SD: 40.6 ± 13.6	34/36	Plaque psoriasis: 30/70 Guttate psoriasis: 20/70 Pustular psoriasis: 20/70	N/A	Mean ± SD: 6.6 ± 5.4	N/A	Newly diagnosed or without systemic treatment ≥2 months before enrolment

[40]	Mean ± SD: 45.6 ± 13.2 Range: 18.0–69.0	10/50	Psoriatic patients; not further clarified	N/A	Mean ± SD: 15.7 ± 9.7 Range: 4.8–64.2	N/A	N/A

[41]	Mean ± SD: 43.8 ± 15.1 Range: 18.0–71.0	21/27	Psoriatic patients; not further clarified	N/A	N/A	N/A	Untreated topically and systematically ≥4 weeks before enrolment

Our study	Mean ± SD: 44.5 ± 15.6	23/9	Active, chronic plaque psoriasis	Excluded	Median: 4.1 Range: 2.2–15.0	Excluded	Untreated topically and systematically ≥1 year before enrolment

Ref. number: reference number; F/M: females/males; N/A: not available; ^*^data retrieved from the abstract, since no full-text file was available.
